# Male Body Odor Affects Emotional State, LH, and Cortisol Secretion in Women of Different Age Groups

**DOI:** 10.3390/brainsci14070721

**Published:** 2024-07-17

**Authors:** Tatiana Laktionova, Ilya Kvasha, Vera Voznessenskaya

**Affiliations:** A.N. Severtsov Institute of Ecology and Evolution, Russian Academy of Sciences, 33 Leninski Prospect, 119071 Moscow, Russia; tatita.laktionova@gmail.com (T.L.);

**Keywords:** olfactory communication, axillary secretions, chemical signal, human, menstrual cycle, emotional state, luteinizing hormone (LH), cortisol

## Abstract

Hormone changes across women’s menstrual cycles may lead to changes in their perceptions of chemical signals and their hormonal responses to these cues. The aim of the present study was to determine the role of menstrual cycle phase in the response to extracts of male axillary secretions (EMAS) in women. We tested healthy reproductive age and premenopausal women (*n* = 29). An EMAS/control solution was applied once every two hours during either the follicular or luteal phase, at which point saliva samples for luteinizing hormone (LH) and cortisol monitoring were collected. LH and cortisol concentrations were analyzed using the enzyme immunoassay (EIA) technique. Positive and Negative Affect Schedule (PANAS) and Visual Analogue Scales (VAS) scores were used to assess the participants’ moods. For the first time, we showed that EMAS may produce opposite effects on LH secretion depending on the menstrual cycle phase of the recipient. We observed a significant increase in the number of LH peaks (*p* = 0.0447) and their amplitudes (*p* = 0.0469) when EMAS was applied during the follicular phase, while the same application in the luteal phase lowered the amplitudes of LH peaks (*p* = 0.0382). For the first time, we showed that EMAS application increased salivary cortisol levels in reproductive age women relative to premenopausal women (*p* = 0.0032). PANAS scores revealed changes in positive and negative affect after EMAS application. Our data indicate the significance of the menstrual cycle phase for EMAS’ effects on LH secretion and mood, but not on cortisol secretion in women.

## 1. Introduction

Body odors are natural components of the environment in a person’s daily life. The past three decades have generated a suite of discoveries supporting, on the one hand, the idea that human olfaction has been underestimated for practically the whole history of investigation [1] and, on the other hand, supporting the potential involvement of body odors in various aspects of interpersonal communication, such as familial bonding, mate selection, and emotional state in humans [2,3]. Additionally, it has been shown that body odor may be processed differently in the brain than other odors [4]. The mechanisms underlying such communication are poorly documented to date.

Human body odors may change the hormonal statuses of women in general, or regulate women’s menstrual cycles in particular [5,6,7]. Apocrine glands in the axillae are currently considered to be the most likely source of human chemical signals, or at least, axillary secretions contribute most to the generation of so-called “body odor” [8]. Preti and colleagues have shown that extracts of male axillary secretions may directly affect luteinizing hormone pulsing and mood in women during the follicular phase of the menstrual cycle [6]. It has been shown that extracts of male axillary secretions affect the length of the menstrual cycle in women with a history of irregular cycle length [5,9]. In premenopausal women, the effects of the application of extracts of male axillary secretions are even more profound than in younger women. It has been shown that idiopathic repeated pregnancy loss was associated with altered olfactory responses to body odor [7]. At the same time, hormone changes across women’s menstrual cycles may lead to changes in their perceptions of chemical signals or other odors and their hormonal responses to these cues [10,11].

Most of the existing studies of body odor’s influence on cortisol secretion were performed on male recipients [12,13]. There are contradictory findings regarding age-related changes in male and female cortisol secretion [14]. At the same time, the start of menopause transition is commonly associated with elevated cortisol secretion [15]. One may expect different responses to body odor, if any, in reproductive age and premenopausal women due to their different initial cortisol levels.

The modulatory effects of male axillary secretions or their components have been addressed in several studies [6,16,17]. The aim of the present study is to determine the role of menstrual cycle phase in the response to male axillary secretions in women.

## 2. Materials and Methods

### 2.1. Participants

A total of 29 healthy heterosexual women were enrolled in the protocol. Informed consent was obtained from all participants. The inclusion criteria for participants were an age between 18 and 55 years old; not taking hormonal pills; and a reported regular menstrual cycle (26–30 days long) for at least 6 months prior testing for reproductive age women (18–45 years old). The exclusion criteria included chronic endocrine or cardiovascular disorders; acute respiratory disorders at the day of testing; pregnancy; and an age under 18 years old.

Women were divided into two groups: 18–45 years old, later referred to as “reproductive age women”, with a mean age of 24.7 ± 5.9 years; and over 45 years old, later referred to as “premenopausal women”, with a mean age of 46.4 ± 3.5 years. For each woman, we had information on the lengths and regularity of her menstrual cycles for 6 months preceding the experiment. Length of menstrual cycles was self-recorded by participants of the study; the beginning of menses was considered the first day of the menstrual cycle. Women in the follicular phase were tested on the 9th or 10th day of their menstrual cycle. Women in the luteal phase were tested 5.3 ± 3.3 days prior to menses, the start of which was later documented.

### 2.2. Extract of Male Axillary Secretions (EMAS)

Axillary secretions were collected over four hours on sterile cotton pads (0.2 × 11 × 9 cm) from 6 healthy heterosexual men of 32.2 ± 4.6 years old using the standard technique [18,19]. For seven days prior to collection, all odor donors were instructed to refrain from using deodorant/antiperspirants and wash/bathe using only plain soap without fragrance. After collecting the secretions, the pads from all the donors were combined and soaked with 70% ethanol (15 mL per pad) (ethanol 96.3% purity, serial # 386710, Ferejn, Moscow, Russia) for 1 h; about 85% of the ethanol extract was recovered. Finally, a masking fragrance (0.005% solution of 2-phenylethanol, CAS Number: 60-12-8, Sigma-Aldrich^®^, St. Louis, MO, USA, Cat. # 77861, ≥99.0% purity) was added to the ethanol extract [19]. A clear 70% ethanol solution scented with the same fragrance was used as an odor-matched control sample. All solutions were stored at −40 °C in aliquots and brought to room temperature before testing. Female participants were unable to distinguish between the scented ethanol extract of male axillary secretions (EMAS) and the control solution by smell. Male donors had no contact and were not familiar with the female participants.

### 2.3. Evaluation of Emotional State of Test Subjects

We evaluated the current emotional state of test subjects using two questionnaires: Positive and Negative Affect Schedule (PANAS) [20] adapted for the Russian population [21] and Visual Analogue Scales (VAS) [22,23]. The questionnaires were printed to fill in by hand.

The PANAS questionnaire consists of a list of 20 adjectives that describe different feelings and emotions. Subjects were asked to note on a five-point Likert scale how they feel with regard to each statement—from “very slightly or not at all” (score 1) to “very much” (score 5). To evaluate total positive or negative affect, we summed up the scores of 10 positive (interested, excited, strong, enthusiastic, proud, alert, inspired, determined, attentive, active) or 10 negative (distressed, upset, guilty, scared, hostile, irritable, ashamed, nervous, jittery, afraid) adjectives.

VAS was presented for 8 adjectives describing mood. They were as follows: attuned to communication, sincere, relaxed, sad, focused, experiencing sexual arousal, energetic, nervous. Participants were instructed to place a mark on a 100 mm scale with anchors “not at all” and “extremely” at the ends in accordance with their current mood.

### 2.4. Procedure

A within-subject, double-blind, repeated-measures design was used in such a way that each participant underwent testing both with the control solution and with EMAS. EMAS or control solution (0.5 mL) were applied to a cotton pad and placed within 15 cm of each woman’s nose for 20 min once every 2 h, during either the follicular or luteal phase of her menstrual cycle. The procedure continued for 8 h (from 10 a.m. to 6 p.m.). Neither experimenter, nor participant were informed about the order in which solutions were applied. None of the women correctly guessed the true nature of the stimuli that were being delivered to their nasal region. Saliva samples were taken every 10 min for reproductive age women (total of 48 samples per woman) using Sali-Caps^®^ sets (IBL, Ratingen, Germany).

The women completed VAS and PANAS four times during the experiment: 15 min after the first application of the control solution at 10 a.m., 15 min before and after the first application of EMAS at 2 p.m., and at the end of the experiment.

Changes in salivary cortisol concentration after EMAS application in a group of reproductive age women were evaluated relative to a group of premenopausal women. For all groups, testing continued for 4 h from 12 a.m. to 4 p.m. and saliva samples were taken every 20 min (total of 12 samples per woman). EMAS or control solution (0.5 mL) were applied to a cotton pad and placed within 15 cm of each woman’s nose once every two hours.

### 2.5. Hormone Measurements and Further Calculations

Saliva samples were stored at −32 °C, thawed at room temperature, and centrifuged before testing. The concentration of luteinizing hormone (LH) was assessed using the enzyme immunoassay (EIA) technique (abx576540, Abbexa, Cambridge, UK) in all saliva samples. After completion of the immunoassay, the absorbance in the wells was measured at λ = 450 nm with a SpectraMax 340PC 384 spectrophotometer (Molecular Devices, Silicon Valley, CA, USA). Data analysis was performed with the software Spectra-MaxSoftware (http://www.moleculardevices.com/pages/software/softmax.html, accessed on 15 May 2024).

The LH baseline was calculated individually for each woman and separately for the control and experimental periods [24,25,26]. To calculate the basal LH level, the mean value of the LH concentration for each woman was added to 1.5 of standard deviation (SD). Values that exceeded the result were *pro tempore* excluded, and the value of the mean + 1.5 SD was calculated again for remaining data. This procedure was repeated until none of the remaining values in the sample exceeded the mean + 1.5 SD. The received remaining value was considered as the basal hormone level for a particular woman in a particular phase.

**Figure 1:**
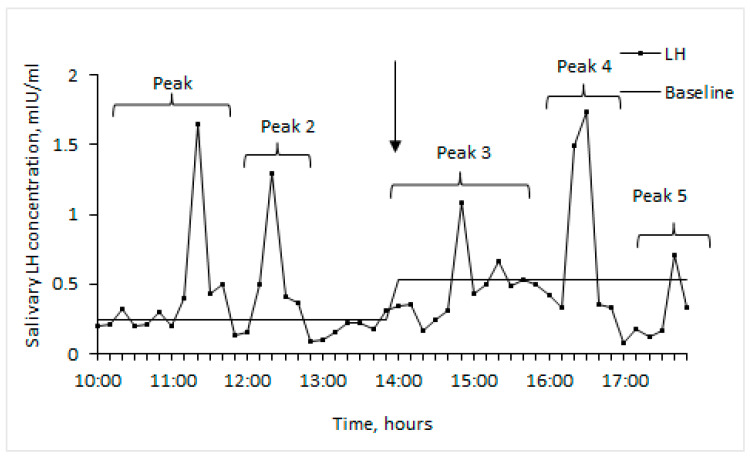
Example of salivary LH pattern of woman tested during follicular phase of menstrual cycle. Arrow indicates beginning of EMAS application (14.00).

We counted the number of LH peaks (values exceeding the basal level) and determined their mean amplitude separately during the control and experimental periods. We also took into account individual differences in the form of the LH peak for each woman and the fact that the number of salivary LH peaks cannot exceed the previously reported number of LH pulses in the blood (on average, one peak per hour in follicular phase and one peak per 2–4 h in luteal phase) [6,27] (Figure 1).

Cortisol was monitored using an EIA technique (EIA Can-C-290, DBC, London, ON, Canada). As cortisol secretion is cyclical in nature, we calculated the average value for two hours during the control period and for two hours during EMAS application to catch the peaks that occur about every 90 min [28], and we used samples that were collected from 12:20 p.m. to 3:40 p.m. from the reproductive age women and premenopausal women. After completion of the immunoassay, the absorbance in the wells was obtained at λ = 450 nm with a SpectraMax 340PC 384 spectrophotometer (Molecular Devices, Silicon Valley, CA, USA). Data analysis was performed with the software SpectraMaxSoftware.

### 2.6. Statistical Analyses

Data analysis was carried out with STATISTICA 8.0 software (StatSoft, Inc., Tulsa, OK, USA) and with R (version 4.3.0).

All data are reported as mean ± SD. All data were confirmed to fit in normal distribution (Shapiro–Wilk test, *p* > 0.05) and to be homoscedastic (Levene’s test, *p* > 0.05) before mixed-design ANOVA was performed, otherwise we used data transformation prior to analysis, such as the non-parametric Wilcoxon signed-rank test or a permutation test with Monte Carlo approximation in R (package lmPerm (https://cran.r-project.org/web/packages/lmPerm/lmPerm.pdf, accessed on 15 May 2024) function aovp(); and package rcompanion (https://cran.r-project.org/web/packages/rcompanion/rcompanion.pdf, accessed on 15 May 2024) function pairwisePermutationTest()). The alpha level for all analysis was set a priori to 0.05.

Analysis of variance for repeated measurements with the within-subject factor treatment (control solution vs. EMAS) and the between-subject factor phase (follicular vs. luteal) was used for comparison of the number of LH peaks.

The LH amplitude data did not fit into normal distribution, which is why we counted the percent of change between mean amplitude during the control period and mean amplitude after the beginning of EMAS application for each test subject, then we performed a Wilcoxon signed-rank test and a one-way ANOVA, followed by a Fisher HSD test.

Cortisol data also did not fit into normal distribution, which is why we counted the percent of change between mean the cortisol level during the control period and the mean cortisol level during EMAS application for each test subject, then we performed a permutation test with a Monte Carlo approximation in R.

## 3. Results

### 3.1. The Parameters of LH Peaks

**Figure 2:**
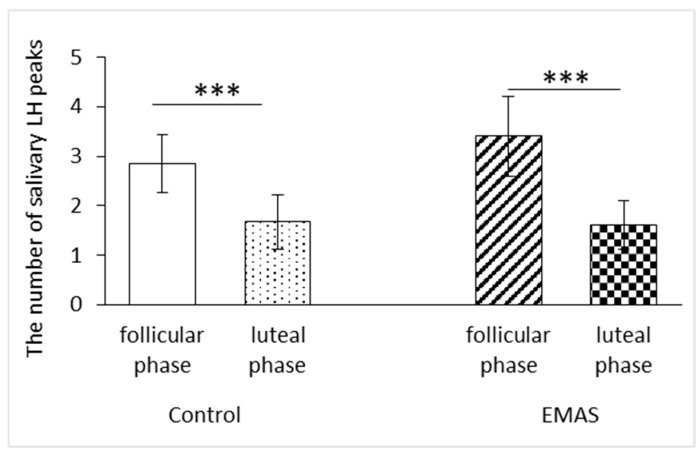
The influence of the menstrual cycle phase on the number of LH peaks (control: *** *p* < 0.001; EMAS: *** *p* < 0.001, *n* = 19, post hoc Fisher LSD test). The data are presented as M ± standard deviation (SD).

We found that the frequency of luteinizing hormone (LH) peaks was affected by the menstrual cycle phases of the test subjects (mixed-design ANOVA, F(1, 17) = 45.37, *p* < 0.001, *n* = 19). In particular, post hoc analysis showed that the number of LH peaks depended on the phase of the menstrual cycle after application of both the control and the extracts of male axillary secretions (EMAS) solutions (*p* < 0.001 and *p* < 0.001, respectively, *n* = 19, post hoc Fisher LSD test) (Figure 2).

**Figure 3:**
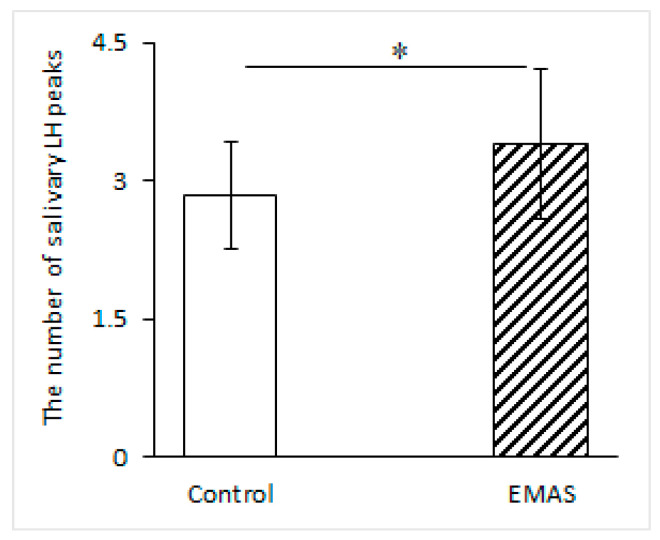
The influence of EMAS application on the number of salivary LH peaks in a group of women during the follicular phase (* *p* = 0.0447, *n* = 10, post hoc Fisher LSD test). The data are presented as M ± SD.

In addition, we observed a significant increase in the number of salivary LH peaks when EMAS was applied to women in the follicular phase of their menstrual cycle compared to the control (*p* = 0.0447, *n* = 10, post hoc Fisher LSD test, effect size d = 0.696, power = 0.45) (Figure 3), which indicates a reduction in the time intervals between adjacent peaks, i.e., an increase in the pulsation of LH. Conversely, in a group of women in the luteal phase of their menstrual cycle, the frequency of LH peaks did not change significantly after EMAS application (*p* = 0.8379, *n* = 9, post hoc Fisher LSD test).

**Figure 4:**
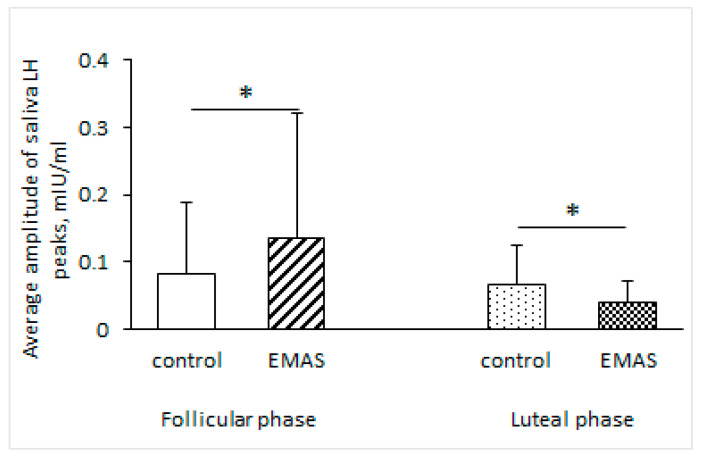
The influence of EMAS application on the average amplitude of salivary LH peaks, depending on the menstrual cycle phases of test subjects (follicular phase: * *p* = 0.0469, *n* = 10; luteal phase: * *p* = 0.0382, *n* = 9, Wilcoxon signed-rank test). The data are presented as M ± SD.

We found a rise in the average amplitude of LH peaks in a group of women in the follicular phase of their menstrual cycle (*p* = 0.0469, *n* = 10, Wilcoxon signed-rank test). We also showed the opposite effect of EMAS application on the average amplitude of LH peaks for the group of women in the luteal phase (*p* = 0.0382, *n* = 9, Wilcoxon signed-rank test) (Figure 4).

### 3.2. Salivary Cortisol

The change in salivary cortisol in reproductive age women did not depend on the phase of the menstrual cycle (mixed-design ANOVA F(1, 17) = 0.92, *p* = 0.3516, *n* = 19, effect size d = 0.70, power = 0.45). However, when comparing groups of reproductive age women and premenopausal women, we found that the change in salivary cortisol after the application of EMAS depended on the age group of the test subjects (permutation test with Monte Carlo approximation, *p* = 0.0032, *n* = 29) (Table 1).

**Figure 5:**
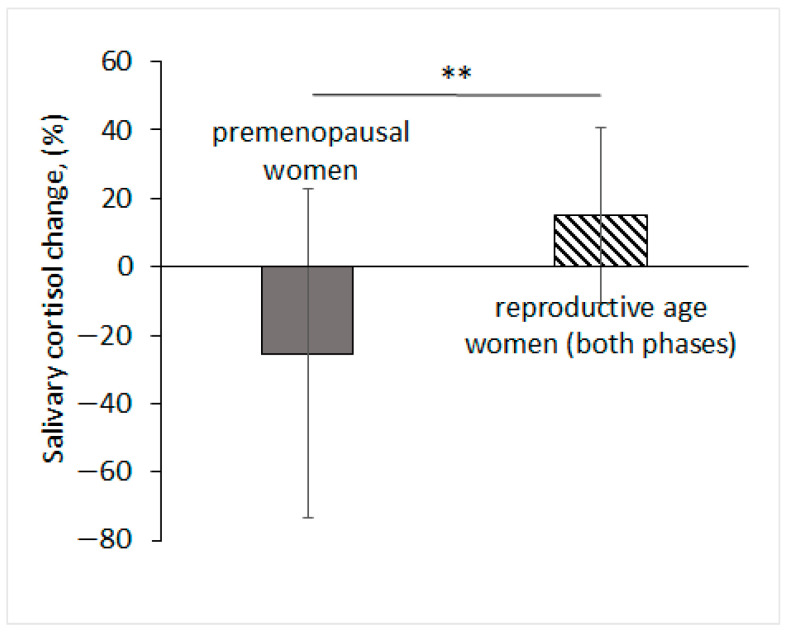
Relative salivary cortisol level changes (in %) after application of EMAS in reproductive age women and in premenopausal women. Data are presented as M ± SD, ** *p* < 0.01, *n* = 29, permutation test with Monte Carlo approximation.

We found that salivary cortisol increased by an average of 15.1% in a group of reproductive age women, and decreased by an average of 25% in a group of premenopausal women after the application of EMAS (Figure 5).

The number of test subjects who reacted with an increase in their level of salivary cortisol after the application of EMAS was significantly higher than could be expected according the natural diurnal changes (follicular phase: *p* = 0.0269, *n* = 10, χ^2^; luteal phase: *p* = 0.0196, *n* = 9, χ^2^). In the group of premenopausal women, the number of test subjects who responded with an increase in salivary cortisol after EMAS application was within the expected level according to diurnal rhythm (*p* = 0.3428, *n* = 10, χ^2^) [28]; in general, we observed a decrease in salivary cortisol in this group.

### 3.3. Emotional State

Mixed-design (repeated measures between and within factors) ANOVA did not reveal significant changes in positive affect 15 min after the beginning of EMAS application, either in the follicular or the luteal phase (mixed-design ANOVA, between-subject factor phase: F(1; 17) = 0.1236, *p* = 0.7294; within-subject factor treatment: F(1; 17) = 0.9082, *p* = 0.3540). At the same time, we observed a decrease in positive affect for the entire time (4 h) of EMAS application (mixed-design ANOVA, between-subject factor phase: F(1; 17) = 0.8690, *p* = 0.3643; within-subject factor treatment: F(1; 17) = 6.2821, *p* = 0.0227), which may also represent the general fatigue of test subjects by the end of the 8 h testing period.

We did not observe any changes in negative affect 15 min after the beginning of application of EMAS compared to the control in women either in the follicular or in the luteal phase (follicular phase: *p* = 0.4652, *n* = 10, Wilcoxon signed-rank test; luteal phase: *p* = 0.4227, *n* = 9, Wilcoxon signed-rank test). We observed an increase in negative affect for the entire time (4 h) of EMAS presentation only in women in the follicular phase (*p* = 0.0464, *n* = 10, Wilcoxon signed-rank test).

**Figure 6:**
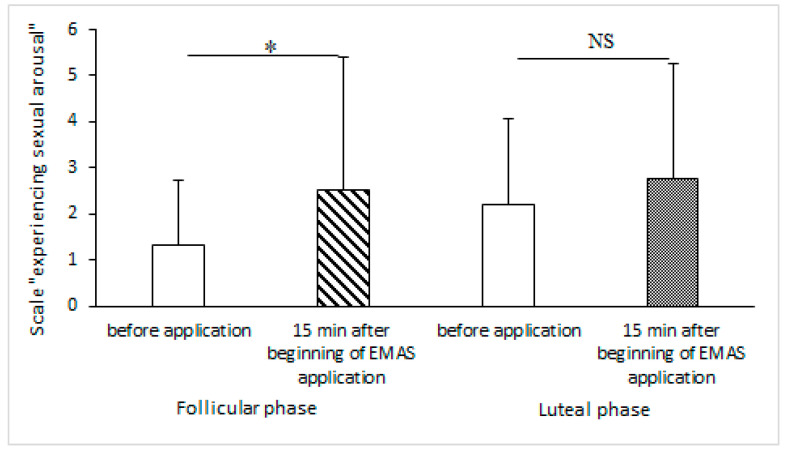
The influence of EMAS application on the parameter “experiencing sexual arousal” in groups of women in the follicular (* *p* = 0.0431, *n* = 10, Wilcoxon signed-rank test) and the luteal (NS—*p* = 0.0587, *n* = 9, Wilcoxon signed-rank test) phases of the menstrual cycle.

VAS scores showed a significant increase in the parameter “experiencing sexual arousal” 15 min after the beginning of EMAS application in women in the follicular phase of their menstrual cycle (*p* = 0.0431, *n* = 10, Wilcoxon signed-rank test), and this parameter showed a tendency to increase in women in the luteal phase of their menstrual cycle (*p* = 0.0587, *n* = 9, Wilcoxon signed-rank test) (Figure 6).

We also observed a short-term increase in the parameter “focused” 15 min after the beginning of EMAS application in a group of women in the follicular phase of the menstrual cycle (*p* = 0.0284, *n* = 10, Wilcoxon signed-rank test), but not in the luteal phase (*p* = 0.9528, *n* = 9, Wilcoxon signed-rank test).

## 4. Discussion

The presentation of extracts of male axillary secretions (EMAS) to healthy heterosexual women could cause a number of different responses, as body odor may provide gender-specific information even if it does not register on a conscious level [4]. These responses may include changes in hormone secretion in both the hypothalamic–pituitary–adrenal and hypothalamic–pituitary–gonadal axes, as well as mood changes and other adaptive reactions in accordance with the context of the signal [3]. With this in mind, we consider the following results of our study as its key findings: (1). The menstrual cycle phase of the recipient determines the hormonal EMAS effects, including an increase in the number of salivary luteinizing hormone (LH) peaks and the average LH peak amplitude after application during the follicular phase, and a decrease in LH amplitude after application during the luteal phase. (2). EMAS application increased salivary cortisol levels in reproductive age women relative to premenopausal women (3). EMAS may affect the emotional states of women, with changes in positive and negative affect by the end of testing, as well as scores of the parameters “experiencing sexual arousal” and “focused” obtained with visual analogue scales (VAS).

To the best of our knowledge, this is the first report of the influence of body odor on LH secretion during the luteal phase of the recipient. We observed a considerable decrease in the average amplitude of LH peaks after EMAS application during the luteal phase, while the number of salivary LH peaks remained the same. Our findings regarding the increase in the number of LH peaks in saliva during the follicular phase are in good agreement with the findings of Preti et al. in blood [6]—extracts of male axillary secretions advance the onset of the next LH peak in women. Moreover, for the first time we found that the average amplitude of salivary LH peaks also increased. Apparently, the observed increase in LH secretion during the follicular phase could be regarded as a possible mechanism for the shortening of abnormally long menstrual cycles, as shown in our previous research [9], since the increase in pulsation of LH may contribute to the acceleration of ovulation. The possible acceleration of ovulation onset due to EMAS application in women with normal cycle lengths suggests a mechanism of cycle lengthening during the luteal phase. Otherwise, it is difficult to explain the constant cycle length [9]. In the present study, we observed a decrease in the average amplitude of LH peaks (a decrease in hormone secretion) after EMAS application during the luteal phase of the menstrual cycle, which may be that exact mechanism of cycle lengthening. We assume that the basis for these results lies in a completely different role that LH performs during the luteal phase compared to the follicular one [27]. The observed phase-sensitive hormonal mechanism may also lie in the basis of the well-documented phenomenon of menstrual synchrony [29]. At the same time, the onset of a new menstrual cycle is manifested primarily by a sharp increase of LH pulse frequency, not a change in their amplitudes [30].

We have designed our experiments with the assumption that the frequency of LH peaks was affected by the phase of the menstrual cycle of each test subject. In particular, during a normal menstrual cycle one can expect a single LH pulse in the blood every 60–90 min during the follicular phase, and one pulse every 3–4 h during the luteal phase [27]. Our current data obtained for saliva have a similar pattern. In addition, we obtained values of LH concentrations in saliva similar to those obtained in other works [31,32].

Since the profile of the LH concentration curve in blood has a specific pulse shape with a pronounced maximum, the software used for the detection of LH pulses in blood—for example, MATLAB module Cluster Analysis, developed by Urban as described in [33]—is based on capturing the sudden change in the sign of the first derivative of the curve from positive to negative. Meanwhile, in saliva, LH peaks have smoother shapes and, as a result, they are not detected as pulses by the specific software described above. Due to the lack of a distinct pulsatile pattern, we have used the baseline method for peak detection of LH [24,25,26].

One can assume the contribution of diurnal changes in LH secretions to the observed effects. Several works are devoted to diurnal changes in LH secretion [34,35,36,37], but their results are inconsistent. One of them shows an absence of circadian rhythms of gonadotropin secretion in women in the early follicular phase [35]. Another shows the presence of some diurnal changes in LH secretion only during the follicular phase [37]. One more study did not report the menstrual cycle phase, and no circadian rhythms for LH secretion were found [36]. So, it is difficult to conclude whether diurnal changes in LH secretion had an impact in our research. At the same time, according to Rossmanith [34], the days of the cycle during which we chose to perform our experiment corresponded to the late follicular and mid-luteal phases of test subjects. For the late follicular phase, there was no increase in LH pulsation in blood during the period of 2 p.m.–6 p.m. when we observed an increase in LH amplitude. For the luteal phase, they observed an increase in LH pulsation from 1 p.m. to 9 p.m., whereas we found a decrease in LH amplitude for the period from 2 p.m. to 6 p.m.

In this work we also evaluated the effect of EMAS application on the secretion of the main human glucocorticoid, cortisol, in women of different age groups. We found that salivary cortisol levels in reproductive age women did not depend on the phase of their menstrual cycle. However, comparing reproductive age and premenopausal women, we found that relative changes in salivary cortisol after EMAS application depended on the age groups of test subjects. There are diurnal changes in salivary cortisol, with the peak that occurs during the first hour after awakening—the so-called cortisol awakening response (CAR)—staying in a consistent range for a single woman during the whole menstrual cycle [38]. During the experiment (12.00–16.00), for every 2 h, salivary cortisol did not change significantly or have a tendency to decline [28,39]. On the contrary, EMAS caused an increase in the levels of salivary cortisol in the reproductive age women by an average of 15.1%. At the same time, cortisol concentrations were within the physiological range both in the experiment and the control, and lower than CAR [38], indicating a possible stimulating effect of EMAS rather than a stressful one.

To the best of our knowledge, this is the first report on the effects of intact male axillary secretions on the levels of cortisol in women. However, for a single component of the axillary secretions—androstadienone—it was shown that its inhalation led to increases in the levels of salivary cortisol in reproductive age women with a mean age of 20–23 y.o. [40].

In premenopausal women, the application of EMAS, on the contrary, lowered the levels of cortisol in saliva compared to the control period by 25%, which corresponded to diurnal changes in human cortisol secretion [28]. Such a different reaction is probably associated with the different hormonal statuses of women of different age groups.

We evaluated the influence of EMAS on the emotional states of women, depending on the phase of the menstrual cycle, using Positive and Negative Affect Schedule (PANAS) and Visual Analogue Scales (VAS) [21,22]. We did not observe any short-term changes in positive or negative affect, but for the entire time (4 h) of EMAS application, we observed a decrease in positive affect for women in both phases, as well as an increase in negative affect in women only in the follicular phase. We suppose that such results may be not only due to EMAS application, but rather represent the general fatigue of test subjects by the end of the 8 h testing period. Meanwhile, VAS scores showed significant short-term increases in the parameter “experiencing sexual arousal” during EMAS application in women in the follicular phase of their menstrual cycle, and a tendency for this to increase during the luteal phase of their menstrual cycle. At first glance, this is not very consistent with the increases in salivary cortisol levels in the same subjects, since sexual arousal can be associated with a decrease in cortisol levels [41]. However, we evaluated a subjective sensation, not the physiological response, and as mentioned before, the increase was significant, but lower than the average value of CAR [38]. The stimulating effect of an increase in salivary cortisol level is also consistent with an increase in the parameter “focused” in the group of reproductive age women in the follicular phase of their menstrual cycle.

Various modulatory effects were shown for male axillary secretions or their components, usually using VAS. For example, male axillary secretions made women more relaxed and less tense [6], while low concentrations of androstadienone made subjects more focused [42]. Activation of the cortical area of the brain, responsible for attention, was also shown when sniffing androstadienone at a sufficiently high concentration exceeding the amount naturally occurring in the axillae [43]. Male axillary secretions can affect the moods of women through the modulation of the serotonin (5-HT) system, namely by a rapid increase in the affinity of the 5-HT transporter without quantitative changes [44]. Although the women in this study were tested only during the follicular phase of their menstrual cycle, the extract of male axillary secretions was applied directly to the area under the nose, and in this case, extract can be absorbed through the skin and enter the bloodstream.

It is possible that more than one compound in axillary secretions can induce endocrine and modulatory changes in human recipients, as far as chemical signals in mammals can be a multicomponent combination of molecules exhibiting a synergistic effect in contrast to the actions of separate ones [45]. The candidates for the role of producing chemical signals from axillary secretions could be among the following: organic unsaturated fatty acids 3-methyl-2-hexenoic acid (3M2H) and 3-hydroxy-3-methyl-hexanoic acid (HMHA); sulfur-containing 3-sulfanyl-3-methyl-hexan1-ol; and volatile steroids, such as 5α-androst-16-en-3α-ol (androstenol) and 4,16-androstadien-3-one (androstadienone), although their concentrations are much lower than those of other compounds [46,47].

To date, most works on the study of components from axillary secretions as chemical signals are devoted to volatile steroids, whereas works on such components as fatty acids are sparse. In a preliminary study by Ferdenzi [46], the perception of the main compound of human sweat odor more characteristic of men, 3-hydroxy-3-methylhexanoic acid (HMHA), was studied in representatives of the European and Madagascar populations. A change in sensitivity to this acid was found to depend on the phase of the menstrual cycle: women in the fertile phase of the cycle (the period of 6 days before ovulation) perceived 3-hydroxy-3-methylhexanoic acid as more intense than women in the non-fertile phase, which made it a possible candidate for being a chemical signal, though further study is required. The changes in LH secretion after EMAS application shown in the current study can be used in future research as a noninvasive bioassay to test the activity of the mentioned components from axillary secretions, which may provide a substantial advance in the field of research into human chemical signals/pheromones. Candidate/s for the role of a human chemical signal should be necessary and sufficient to recreate the original activity of axillary secretions with the original bioassay at naturally occurring concentrations [45].

There are two hypotheses on how individuals assess potential mating partners via telereceptive senses such as vision, olfaction, and hearing. According to the backup signals hypothesis [48,49,50], multimodal cues provide redundant information, whereas the multiple messages hypothesis [48] suggests that different modalities provide independent and distinct information about an individual’s mating-related quality. Our results regarding the significant effect of EMAS application on hormone secretion fall in better with the multiple messages hypothesis. They are also in good agreement with meta-analysis [51] that showed that body odor may provide distinct and non-redundant information about an individual’s mating-related qualities as opposed to that accessible through either visual or vocal cues.

## 5. Conclusions

The results of our study elucidate the phase-sensitive hormonal mechanism of the significant effects of male body odor on menstrual cycle in women. Using a noninvasive method, we demonstrated, for the first time, that male body odor: (1) may increase not only the number of salivary LH peaks, but also their amplitudes during the follicular phase of the menstrual cycle; (2) may decrease the amplitudes of LH peaks (the decrease in secretion) during the luteal phase of the menstrual cycle; (3) may increase cortisol secretion in reproductive age women relative to premenopausal women. We have validated a noninvasive convenient bioassay for the further testing of single compounds from male body odor that are potentially responsible for the observed effects. Since no such compounds have been identified yet, the availability of such a bioassay may substantially advance research in the field of human chemical communication.

## Figures and Tables

**Table 1:** Results of permutation test with Monte Carlo approximation.

Component 1:					
	Df	R Sum Sq	R Mean Sq	Iter	Pr(Prob)
Age Group	1	10,697	10,696.9	5000	0.0032 **
Residuals	27	32,726	1212.1		

Signif. codes: ‘**’ 0.01 ‘.’ 0.1 ‘ ’ 1.

## Data Availability

The original contributions presented in the study are included in the article; further inquiries can be directed to the corresponding author.
